# Protein-truncating variants and deletions of *SHANK2* are associated with autism spectrum disorder and other neurodevelopmental concerns

**DOI:** 10.1186/s11689-025-09600-0

**Published:** 2025-04-30

**Authors:** Hailey Silver, Rori Greenberg, Paige M. Siper, Jessica Zweifach, Renee Soufer, Mustafa Sahin, Elizabeth Berry-Kravis, Latha Valluripalli Soorya, Audrey Thurm, Jonathan A. Bernstein, Alexander Kolevzon, Dorothy E. Grice, Joseph D. Buxbaum, Tess Levy

**Affiliations:** 1 Seaver Autism Center for Research and Treatment, Icahn School of Medicine at Mount Sinai, New York, NY 10029 USA; 2 Department of Psychiatry, Icahn School of Medicine at Mount Sinai, New York, NY 10029 USA; 3 The Mindich Child Health and Development Institute, Icahn School of Medicine at Mount Sinai, New York, NY 10029 USA; 4 Department of Neurology, Rosamund Stone Zander Translational Neuroscience Center, Boston Children’s Hospital, Harvard Medical School, Boston, MA 02115 USA; 5 F.M. Kirby Neurobiology Center, Boston Children’s Hospital, Harvard Medical School, Boston, MA 02115 USA; 6 Developmental Synaptopathies Consortium, Rare Disease Clinical Research Network, Boston, USA; 7 Department of Pediatrics, Rush University Medical Center, Chicago, IL 60612 USA; 8 Department of Neurological Sciences, Rush University Medical Center, Chicago, IL 60612 USA; 9 Department of Anatomy and Cell Biology, Rush University Medical Center, Chicago, IL 60612 USA; 10 Department of Psychiatry & Behavioral Sciences, Rush University Medical Center, Chicago, IL 60612 USA; 11 Neurodevelopmental and Behavioral Phenotyping Service, National Institute of Mental Health, National Institutes of Health, Bethesda, MD 20892 USA; 12 Department of Pediatrics, Stanford University School of Medicine, Stanford, CA 94304 USA; 13 Department of Pediatrics, Icahn School of Medicine at Mount Sinai, New York, NY 10029 USA; 14 Division of Tics, OCD and Related Disorders, Icahn School of Medicine at Mount Sinai, New York, NY 10029 USA; 15 Friedman Brain Institute, Icahn School of Medicine at Mount Sinai, New York, NY 10029 USA; 16 Department of Genetics and Genomic Sciences, Icahn School of Medicine at Mount Sinai, New York, NY 10029 USA; 17 Department of Neuroscience, Icahn School of Medicine at Mount Sinai, New York, NY 10029 USA

**Keywords:** SHANK2, Autism spectrum disorder, Developmental delay, Intellectual and developmental disability, Phenotype

## Abstract

**Background:**

SHANK2 disorder is a rare neurodevelopmental disorder caused by a deletion or pathogenic sequence variant of the *SHANK2* gene and is associated with autism spectrum disorder (ASD), intellectual disability (ID), and developmental delay. To date, research in *SHANK2* has focused on laboratory-based in vivo and in vitro studies with few prospective clinical studies in humans.

**Methods:**

A remote assessment battery was comprised of caregiver interviews with a psychiatrist, psychologists, and a genetic counselor, caregiver-reports, and review of records. Results from this cohort were reported using descriptive statistics. An age-matched sample of participants with *SHANK3* haploinsufficiency (Phelan-McDermid syndrome, PMS) was used to compare adaptive behavior between the two groups.

**Results:**

All ten participants demonstrated delays in adaptive behavior, with most motor skills preserved and a weakness in communication. According to parent report, 90% of participants carried a formal diagnosis of ASD, 50% of participants carried a diagnosis of attention-deficit/hyperactivity disorder (ADHD), and mild-to-moderate developmental delays were noted. Sensory hyperreactivity and seeking behaviors were more pronounced than sensory hyporeactivity. Medical features included hypotonia, recurrent ear infections, and gastrointestinal abnormalities. No similar facial dysmorphic features were observed. Compared to PMS participants, individuals with SHANK2 disorder had significantly higher adaptive functioning.

**Conclusions:**

Consistent with previous studies of SHANK2 disorder, these results indicate mild to moderate developmental impairment. Overall, SHANK2 disorder is associated with developmental and adaptive functioning delays, high rates of autism, including sensory symptoms and repetitive behaviors, and ADHD. This study was limited by its remote nature, diverse age range, and the homogeneous racial and ethnic sample. Future studies should examine larger, diverse cohorts, add cognitive testing, capture longitudinal data, and include in-person assessments.

## Background

Over the past decade there has been a significant increase in the use and utility of genetic testing in cases of intellectual and developmental disorders [1–7]. To date, more than 250 high confidence genes associated with autism spectrum disorder (ASD) [7–10] and other neurodevelopmental disabilities such as intellectual disability (ID) [11–14] have been identified. *SHANK2* is one such gene.

*SHANK2* is part of the SHANK gene family, which organizes intermediate scaffolding proteins at excitatory synapses and are categorized as master scaffolding proteins [15] responsible for the structural integrity of dendritic spines in the postsynaptic density [16]. The *SHANK* family is involved in activity at the postsynaptic sites of excitatory synapses in the brain. Loss of function of *SHANK2* is associated with synaptic dysfunction [16]. Other proteins in the SHANK gene family include *SHANK1* and *SHANK3*. *SHANK3* deficiency leads to a neurodevelopmental disorder known as Phelan-McDermid syndrome (PMS). PMS is associated with high rates of intellectual and developmental disability (IDD), autism, sensory symptoms, hypotonia, neuropsychiatric symptoms, and a number of medical comorbidities [17–21].

The knockout of *SHANK2* in mice has been used to create a model for neuropsychiatric symptoms including ASD [22–29]. There are several variants of SHANK2 mouse models with phenotypic differences based on which cell types the *SHANK2* deletion occurs in and the exon knockout location [28–31]. Observed phenotypes include enhanced fear causing behavioral inflexibility [22, 27], hyperactivity [30–31], repetitive grooming [30–31] or jumping behaviors [25], and sensory hyperreactivity [24]. Some previous preclinical studies experimented with molecular interventions to compensate for the loss of *SHANK2*. For example, improvement in social interaction in a SHANK2 mouse model was reported after glutamate modulation [25]. Similarly, Chung and colleagues [26] observed that when early development N-methyl-D-aspartate glutamate receptor (NMDAR) hyperfunction was suppressed, later NMDAR hypofunction and ASD-like behaviors decreased. In addition to autism, the *SHANK2* gene has also been associated with schizophrenic behaviors [32] and decreased bone mass [33] in animal models.

To date, research in *SHANK2* has focused on in vivo and in vitro studies with limited clinical studies in humans. Prior clinical research includes small cohort studies of one to three participants [34–36] and gene discovery studies where participants with SHANK2 were identified [37–42]. Two recent publications reviewed the literature and collated these small cohorts with their own as a comparison [43–44]. The largest was a literature review of 13 previously identified cases along with one additional participant [44]. When reported, all patients had mild to moderate ID and language delays, 92% had ASD or autism traits, and 20% had difficulties with attention or sleep disorders [44]. Outside of these distinctions, there was a noted variability in the understanding of the SHANK2 clinical phenotype from smaller cohort studies [44], indicating the need for additional studies in larger cohorts. No studies to date have compared phenotypes of SHANK2 disorder with that of PMS (*SHANK3* haploinsufficiency).

Previous literature relied on case reports and chart review with very limited prospective phenotyping. Here, we present the results of a prospective, remote-based clinical phenotyping study in ten participants with SHANK2 disorder. This study was the first to include a larger, systematically evaluated cohort of individuals with the disorder. We included assessments that covered a wide range of domains and phenotypic features in accordance with the recommendations from AlMail and colleagues [45], which outlined the recommended battery for reporting a new rare genetic disorder. Following the success of a remote battery used at our Center to phenotype CHAMP1 syndrome [46], this battery of assessments was administered remotely as well. This approach removed barriers to participation and allowed us to include individuals from multiple countries.

## Methods

### Participants

**Fig. 1:**
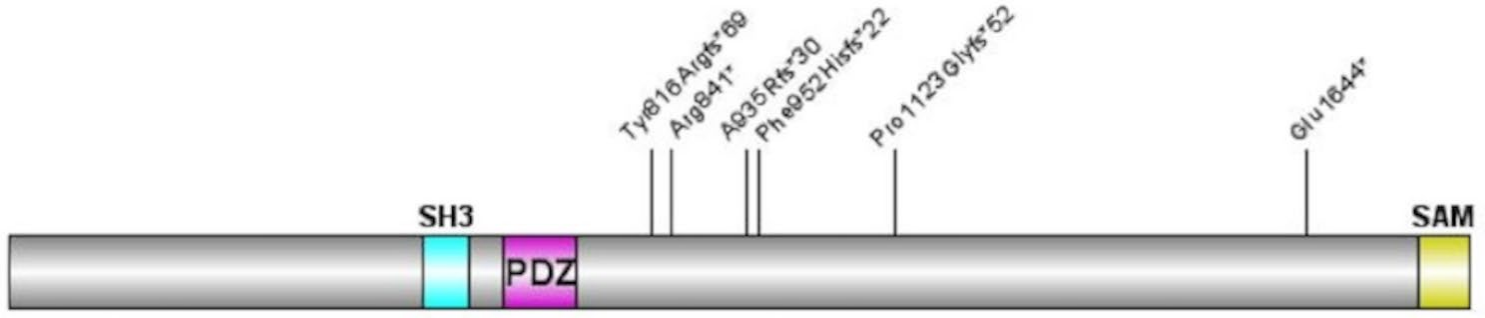
SHANK2 Genetic Landscape *Legend*: Individuals’ *SHANK2* variants mapped onto the gene, located on chr11:70,313,959 − 70,963,623 (hg19). Participants with deletions all carried deletions within the *SHANK2* gene (see Table 1)

Ten participants between the ages of 3 to 25 years (M_age_ = 9.7 ± 6.7) enrolled in this study. All participants were white, non-Hispanic, and most were female (*n* = 7). Study recruitment was advertised by the Seaver Autism Center and by the SHANK2 Foundation and caregivers contacted the study team directly to participate. Inclusion criteria included a confirmed pathogenic or likely pathogenic deletion or sequence variant in *SHANK2* and the ability for a caregiver to read and understand English. All eligible participants who contacted the study team were included in the study. The caregiver’s primary language was not assessed. Table 1; Fig. 1 provide the genetic landscape for this sample. This study was approved by the Program for the Protection of Human Subjects at the Icahn School of Medicine at Mount Sinai.

**Table 1:** SHANK2 genetic landscape

**Sequence Variants**		
**Coding DNA Change**	**Amino Acid Change**	**Inheritance**
c.2802dupC	p.Ala935Argfs*30	de novo
c.2826_2853dup	p.Phe952Hisfs*22	de novo
c.2445_2446delGT	p.Tyr816Argfs*69	de novo
c.3364_3365dup	p.Pro1123Glyfs*52	unknown
c.4930G > T	p.Glu1644*	de novo
c.2521 C > T	p.Arg841*	de novo
**Copy Number Variants (Deletions)**		
**Start Coordinate**	**End Coordinate**	**Inheritance**
70,419,765	70,606,442	unknown
70,384,774	70,613,589	unknown
70,423,040	70,685,140	unknown
70,317,016	70,535,034	de novo

*Legend*: Sequence variants in *SHANK2* in the cohort, mapped onto transcript NM_012309.5. Deletions of *SHANK2* are reported in hg19 start and end coordinates. All deletions only include *SHANK2*

### Materials

To facilitate the study of this ultra-rare disorder, a remote phenotypic protocol was applied [46]. All caregiver interviews were conducted using HIPAA-compliant Zoom. Caregiver questionnaires were completed via REDCap or on scoring platforms provided directly by the publisher.

Skill development and loss was assessed by the Early Skills Attainment and Loss caregiver interview, a measure focusing on regression [47]. This instrument measured Language, Motor, Social, and Adaptive skill attainment and loss. If a skill was attained, the interviewer either asked if the skill was attained by age 1, or the specific month at which it was attained. Skill loss was defined as the discontinuation of a skill that was previously obtained and used consistently for at least 3 months. Developmental milestones were analyzed by comparison to Center for Disease Control (CDC) guidelines, as reported in Zubler et al. [48].

Psychiatric and medical history were assessed by a psychiatrist (DEG) through a structured caregiver interview. Participants were present for a portion of the interview for virtual observation. Dysmorphology was assessed by caregiver report using a standardized list of dysmorphic features and analysis of front and side profile photos by a certified genetic counselor and trainee (TL, RG).

The Developmental Profile, Fourth Edition (DP-4; [49]), is a caregiver interview that obtained estimates of developmental functioning across Physical, Adaptive Behavior, Social-Emotional, Cognitive, and Communication domains. Standard scores could not be obtained for one participant who was out of the normed age range.

The Vineland Adaptive Behavior Scales, 3rd Edition, Comprehensive Interview (Vineland-3; [50]), assessed adaptive behavior through caregiver interview across Communication, Daily Living Skills, Socialization, and Motor domains. Standard scores are available across all ages, but only for participants nine and younger on the Motor domain.

Sensory symptomatology was assessed with the Sensory Assessment for Neurodevelopmental Disorders (SAND; [51]) for Hyperreactivity, Hyporeactivity, and Seeking behaviors across visual, tactile, and auditory modalities; and with the Sensory Profile Caregiver Questionnaire (SP; [52]) assessing the following domains: Auditory, Visual, Touch, Taste/Smell, Activity Level, Body Position, Emotional/Social, and Movement Processing across four quadrants: Low Registration, Seeking, Sensitivity, and Avoidance. Due to the remote nature of the study, only the interview portion of the SAND was conducted with the caregiver, and the observation was omitted.

The Child/Adult Behavior Checklist (CBCL/ABCL; [53]) assessed caregiver-reported behaviors. Domains included Syndrome Scales, Internalizing, Externalizing, Total Problems, and DSM-Oriented Scales. Three caregivers completed the CBCL for ages 1.5 to 5, six completed the CBCL for ages 6 to 18, and one caregiver completed the ABCL for ages 18 and older. Subdomains assessed differed by form completed.

The Aberrant Behavior Checklist (ABC; [54]) is a caregiver report questionnaire that assessed maladaptive behaviors within five domains: Irritability, Lethargy, Stereotypy, Hyperactivity, and Inappropriate Speech. The frequency and severity of repetitive behaviors was measured by caregiver report using the Repetitive Behavior Scale, Revised (RBS-R; [55]) across the following scales: Stereotyped, Self-Injury, Compulsive, Ritualistic, Insistence on Sameness, and Restricted Behaviors.

Quality of Life and Caregiver Concerns were assessed using a Visual Analogue Scale (VAS) where caregivers list their top three concerns for the participant and rate how concerned they were about these items from 1 to 100; and using the Child and Family Quality of Life, Second Edition (CFQL; [56]), capturing the caregiver’s perspective of their child’s and their own quality of life. It includes the following domains: Child, Family, Caregiver, Financial, Social, Relationship, Coping, and Changes in Quality of Life.

The battery included validated and unvalidated assessments. Most of the assessments were published validated measures including the DP-4, Vineland-3, SP, CBCL/ABCL, ABC, RBS-R, and CFQL. One published standardized assessment, the SAND, includes both a caregiver interview and direct observation of the child, however, due to the remote nature of the study, the direct observation was omitted; therefore, only raw scores were produced from the interview. Three assessments were not validated but have been used in similar populations including the Early Skills, the structured psychiatric evaluation, and the VAS. The assessments selected were recently successfully used in a prior remote battery of an IDD sample [46] and were considered appropriate for remote use.

### Comparison to SHANK3 cohort

Overall adaptive functioning in the SHANK2 cohort was compared to an age-matched group of individuals with PMS (*N* = 62, M_age_ = 9.64, SD = 5.52, range = 3–24 years) which included a subset of individuals participating in a natural history study through the Developmental Synaptopathies Consortium.

## Results

### Early skill development

Early skill development and loss, assessed by the early skill attainment and loss survey and clinician interview, is summarized in Tables 2 and 3.

**Table 2:** Early skills achievement I

Skill	*N* Achieved	*N* Achieved by age 1	*N* Achieved after age 1	Typical Achievement [48]
**Social Skills**				
Respond to name	10	3	7	9 months
Social smile	9	8	1	2 months
Point at object or event	8	1	7	18 months
Eye contact	10	7	3	2 months
Wave goodbye	9	2	7	12 months
Show objects to others	9	0	9	15 months
**Motor**				
Reach for an object	10	8	2	6 months

**Table 3:** Early skills achievement II

Skill	*N* Achieved	Range (months)	Mean ±SD Median (IQR)	Typical Achievement [48]
**Motor**				
Roll over	9	4–18	7.7 ± 4.6 months 6.0 (5.0–8.0) months	6 months
Sit without support	10	6–13	8.2 ± 2.4 months 7.5 (7.0–8.0) months	9 months
Crawl	9	8–16	10.9 ± 2.8 months 10.0 (9.0–13.0) months	n/a
Take independent steps	10	12–22	16.5 ± 3.5 months 16.3 (13.3–19.5) months	15 months
**Language**				
Babble	9	6–36	13.0 ± 9.8 months 9.0 (8.0–10.0) months	9 months
Single words	9	14–42	2.4 ± 1.0 years 2.0 (1.5–3.3) years	15–18 months
Two-word phrases	8	30–62	3.8 ± 1.1 years 3.8 (2.9–4.8) years	24 months
Fluent speech	7	48–96	5.7 ± 1.4 years 5.5 (4.8–6.5) years	36–48 months
**Toileting**				
Bladder control	7	36–288	7.5 ± 7.7 years 4.0 (3.7-7.0) years	∼ 36 months*
Bowel control	7	36–120	4.8 ± 2.5 years 4.0 (3.4-5.0) years	

*Legend*: Tables separated by skill domain indicating the number of participants who achieved each skill and when with reference to the skill’s recommended attainment age as published by Zubler et al. [48]. *Milestone range obtained separately from “Five dos and don’ts of potty training your toddler” [57]

Language milestones were delayed in all participants. Seven participants used full sentences or phrases of three or more words. Of those, five used full complex sentences with appropriate grammar and two used phrases and combined more than three words. One five-year-old participant used two-word phrases; one three-year-old used single words; and one three-year-old was babbling but had not yet said a first word. Importantly, participants who had not yet developed fluent speech were below the average age of achievement in this cohort (∼ 5y). Articulation problems were reported in six of ten participants.

Using the CDC guidelines for skill development, which are set at the developmental age of the 75th percentile [48], two of the three motor milestones with CDC recommended checkpoints were delayed on average across participants. Specifically, 44% of participants were delayed rolling over, 20% were delayed in sitting without support, and 60% were delayed taking independent steps. No loss of motor skills was reported.

Seven participants obtained bladder and bowel control. Delays were present for both. On average, bowel control was obtained before bladder control. Skill loss was reported in one participant. The oldest participant lost bladder control at 24.5 years and had not regained the skill, though bowel control remained. The caregiver reported these and other declines during the COVID-19 pandemic and attributed them to a lack of structure and engagement in daily activities. Daily living skills generally improved when returning to a structured schedule.

### Psychiatric & medical history

Nine participants carried a Diagnostic and Statistical Manual of Mental Disorders, Fifth Edition (DSM-5; [58]) diagnosis of ASD. Deficits in social/emotional reciprocity, nonverbal communication, and stereotyped behaviors were reported in all nine of those participants. Seven caregivers reported symptoms of attention-deficit and hyperactivity, including six with inattention and impulsivity and five with hyperactivity. Five participants carried a diagnosis of attention-deficit/hyperactivity disorder (ADHD): one with inattentive type, one with hyperactive-impulsive type, two with combined type, and the remaining participant was uncategorized. One individual was diagnosed with obsessive compulsive disorder (OCD). Previous diagnoses of ID and global developmental delay were not directly ascertained.

Five participants took psychotropic medications in the past and two were actively taking medication. Of those on medication at the time of the evaluation, one was on guanfacine for ADHD and the other was on both trazadone and clonidine for sleep. Regarding prior medications, methylphenidate for ADHD, two unnamed ADHD stimulants, and aripiprazole were all reportedly stopped due to side effects. Fluoxetine was prescribed for OCD but was stopped due to ineffectiveness. Lithium was tried for mood and discontinued in one participant due to challenges in achieving appropriate blood levels, and one participant lisdexamfetamine and guanfacine extended release were both stopped due to intolerability.

Medical history is summarized in Table 4. Four participants had tympanostomy. Two participants had a history of hearing problems reported: one due to increased fluid and another due to multiple ear infections. Both participants’ hearing returned to normal after correcting these issues. One participant had a single febrile seizure at age 2 reported, but no presence of subsequent seizures.

**Table 4:** Medical history

Symptom	*N*	Percentage
Hypotonia	7/10	70%
Otitis Media	6/9	67%
GI Tract Abnormalities	5/10	50%
Constipation	5/10	50%
Gastroesophageal Reflux Disease (GERD)	1/10	10%
Tympanostomy	4/9	44%
Gait Abnormalities	4/10	40%
Toe walking	3/10	30%
Orthotics for ankle support	1/10	10%
Knee hyperextension	1/10	10%
Sleeping Problems	4/10	40%
Difficulty falling asleep	4/10	40%
Difficulty staying asleep	3/10	30%
Waking early	1/10	10%
Feeding Issues	3/10	30%
Chewing	2/10	20%
Swallowing	2/10	20%
Difficulty latching	1/10	10%
Vision Abnormalities	2/10	20%
Amblyopia	1/10	10%
Hyperopia	1/10	10%
Strabismus	1/10	10%
Hearing Abnormalities, since corrected	2/10	20%
Allergies	2/10	20%
Psoriasis/Dermatitis	1/9	11%
Febrile Seizure (1x)	1/10	10%

*Legend*: Reported medical history from most to least frequent. *One caregiver did not answer all questions on medical history form

### Dysmorphology

Dysmorphic features were reported by caregivers and identified through images provided. Most dysmorphic features were only noted in one participant each. Four participants had a high frontotemporal hairline. Three participants had full cheeks, two had an overbite, and two had a long philtrum. The following features were present in one participant each: micrognathia, bulbous nose, full lips, hyperextensibility, sacral dimple, and fifth finger clinodactyly. Similar facial gestalts were not seen in the cohort.

### Cognitive and adaptive functioning

DP-4 (Table 5) Physical domain standard scores ranged from 47 (delayed) to 101 (average). Adaptive Behavior standard scores ranged from 53 (delayed) to 88 (average). Social-Emotional Skills standard scores range from 57 (delayed) to 78 (below average). The Cognitive subdomain standard scores ranged from 47 (delayed) to 77 (below average). The Communication subdomain standard scores ranged from 51 (delayed) to 79 (below average).

**Table 5:** Developmental Profile-4 results

Domain	Mean (SD)	Qualitative Descriptor	Participants Below 70 (percentage)
Physical	78.2 (20)	Below Average	44%
Adaptive Behavior	68.7 (12.1)	Delayed	56%
Social-Emotional Skills	68.9 (7.8)	Delayed	44%
Cognitive	65.4 (11.3)	Delayed	44%
Communication	68.7 (8.7)	Delayed	44%

*Legend*: DP-4 results by mean, standard deviation, and qualitative descriptor of the mean. Percentage of the 9 participants below a score of 70 represent those two standard deviations below the normative mean, indicating significant delays

Vineland-3 Adaptive Behavior Composite standard scores ranged from 20 to 72 (Table 6). The Communication domain had the lowest average standard score among all domains. Six participants had significantly higher subdomain scores in the Receptive subdomain compared to the Expressive subdomain (> 1SD difference), two participants had no difference, and one participant had a significantly higher Expressive subdomain scores than Receptive. All participants were reported to understand at least 50 words and eight participants said at least 50 words and used phrases with a noun and verb. Six participants were reported to identify all letters in lower and upper case, five could read at least 10 words, four could read simple sentences out loud, and two could read simple stories. Four participants could copy their own first name, three could copy phrases or sentences, and two write at least 20 words from memory including simple sentences.

**Table 6:** Vineland-3 results

Domain/Subdomain	Range	Mean (SD)	Qualitative descriptor
**Adaptive Behavior Composite**	**20–72**	**59.1 (15.34)**	**Low**
**Communication**	**20–62**	**47.8 (12.7)**	**Low**
Receptive	1–10	6.2 (3.36)	Low
Expressive	1–8	3.6 (2.88)	Low
Written	1–10	7.3 (2.63)	Low
**Daily Living Skills**	**20–85**	**65.0 (18.37)**	**Low**
Personal	1–13	6.5 (4.22)	Low
Domestic	1–18	10.2 (4.98)	Moderately Low
Community	1–10	7.4 (2.72)	Low
**Socialization**	**20–81**	**63.3 (18.84)**	**Low**
Interpersonal Relationships	2–15	7.6 (3.60)	Low
Play and Leisure	1–12	8.3 (3.59)	Low
Coping Skills	7–14	9.6 (2.59)	Moderately Low
**Motor (** ***n*** ** = 6)**	**70–89**	**76.0 (7.04)**	**Moderately Low**
Gross Motor	8–14	11.0 (2.37)	Moderately Low
Fine Motor	8–14	9.83 (2.40)	Moderately Low

*Legend*: Vineland results by domain (bold) and subdomain. Scores reported by range, mean, and standard deviation include domain standard scores (*M* = 100, *SD* = 15) in bold, and subdomain v-scale scores (*M* = 15, *SD* = 3)

Regarding Daily Living Skills, Domestic Skills were a relative strength with the highest average subdomain score compared to average Personal and Community subdomain scores. In terms of specific skills, eight participants fed themselves without spilling, five could dress themselves and correctly put on and fasten their own shoes. Five participants brush their teeth independently. While seven had bladder and bowel control, only four used the bathroom completely independently, and only two shower or bathe independently. In the Socialization domain, scores across Interpersonal Relationship, Play and Leisure, and Coping Skills fell within the low range. Finally, standard scores for the Motor domain were calculated for the six participants within the age range to calculate normed scores. Motor skills represent a relative strength of this cohort compared to performance on other domains, however, both fine and gross motor skills remain delayed relative to age expectations.

### Sensory symptomatology

Sensory symptoms were evaluated using the SAND interview and the SP. Sensory Seeking behaviors were most common (7.8 ± 4.6), followed by Hyperreactivity (5.6 ± 2.7), and Hyporeactivity (3.3 ± 3.5). The most commonly reported Sensory Seeking behaviors were creating sounds outside of functional play (6/10), seeking pressure including pushing objects to self or mouthing (6/10), peering at or inspecting parts of toys near their eyes (5/10), fascination with certain textures (5/10), feeling textures repeatedly (5/10), and fascination with certain sounds (5/10). The most commonly reported sensory Hyperreactivity behaviors were startling or being bothered by certain sounds (6/10) and putting their hands over their ears (5/10). The most commonly reported sensory Hyporeactivity behaviors were under-responsiveness to bright or flickering lights (4/10) and under-responsiveness to temperature and/or pain (4/10).

On the SP, Low Registration scores (51.5 ± 7.1) indicated a definite difference in sensory processing, with nine participants scoring in the definite and one in the probable difference range. Sensation Seeking scores (90.7 ± 12.3) indicated a definite difference with six participants falling in the definite and one in the probable difference range. Sensory Sensitivity scores (75.6 ± 8.8) indicated a probable difference in sensory processing, with four participants in the definite and two in the probable difference range. Sensation Avoiding scores (106 ± 9.5) indicated a probable difference, with three participants scoring in the definite and five in the probable difference range.

### Behavioral symptomatology

Average T-scores from the CBCL indicated that Withdrawn Behaviors and Attention Problems fell in the clinical range. Autism Spectrum, ADHD Symptoms, Activity Participation, School Participation, Social Participation, Thought Problems, and Critical Items all fell in the borderline range. Of the ten participants, 50% were in the clinical range for Attention Problems and ADHD Symptoms and of the seven assessed for thought problems, 57% were in the clinical range. Those in the clinical range matched the prevalence of participants who met criteria for ADHD when assessed in the psychiatric evaluation. However, thought problems were not noted or brought up by caregivers as a concern during the psychiatric evaluation and when analyzing individual items, the most commonly endorsed items in the Thought Problems domain were related to ritualistic and repetitive behaviors (RRBs) and likely reflect autism symptomology.

Average scores on the ABC domains were 9.9 ± 6.8 for Irritability (45 maximum score), 3.1 ± 3.2 for Social Withdrawal (48 maximum score), 3.3 ± 2.7 for Stereotypy (21 maximum score), 15.3 ± 11.3 for Hyperactivity (48 maximum score), and 2.8 ± 1.9 for Inappropriate Speech (12 maximum score). At the individual item level, nine caregivers reported distractibility. Eight reported temper tantrums, inattention, disturbing others, impatience, and impulsivity. Seven caregivers also reported stamping feet or slamming doors, disrupting group activities, repetitive speech, and stereotyped behaviors.

On the RBS-R, difficulty with transitions was endorsed by all ten caregivers. Seven caregivers endorsed sensory features and specific interests. The Average Total Score was 20.4 ± 14.3 with a range between 1 and 47 points out of a maximum total of 129. The subscale scores were as follows: Stereotyped Behavior (3.7 ± 4.2) out of 18 points, Insistence on Sameness (6.2 ± 3.9) out of 33 points, Ritualistic Behavior (3.1 ± 3.2) out of 18 points, Restricted Behaviors (1.9 ± 1.7) out of 12 points, Compulsive Behavior (3.1 ± 3.9) out of 24 points, and Self-Injurious Behaviors (2.4 ± 2.9) out of 24 points.

### Quality of life and caregiver concerns

**Fig. 2:**
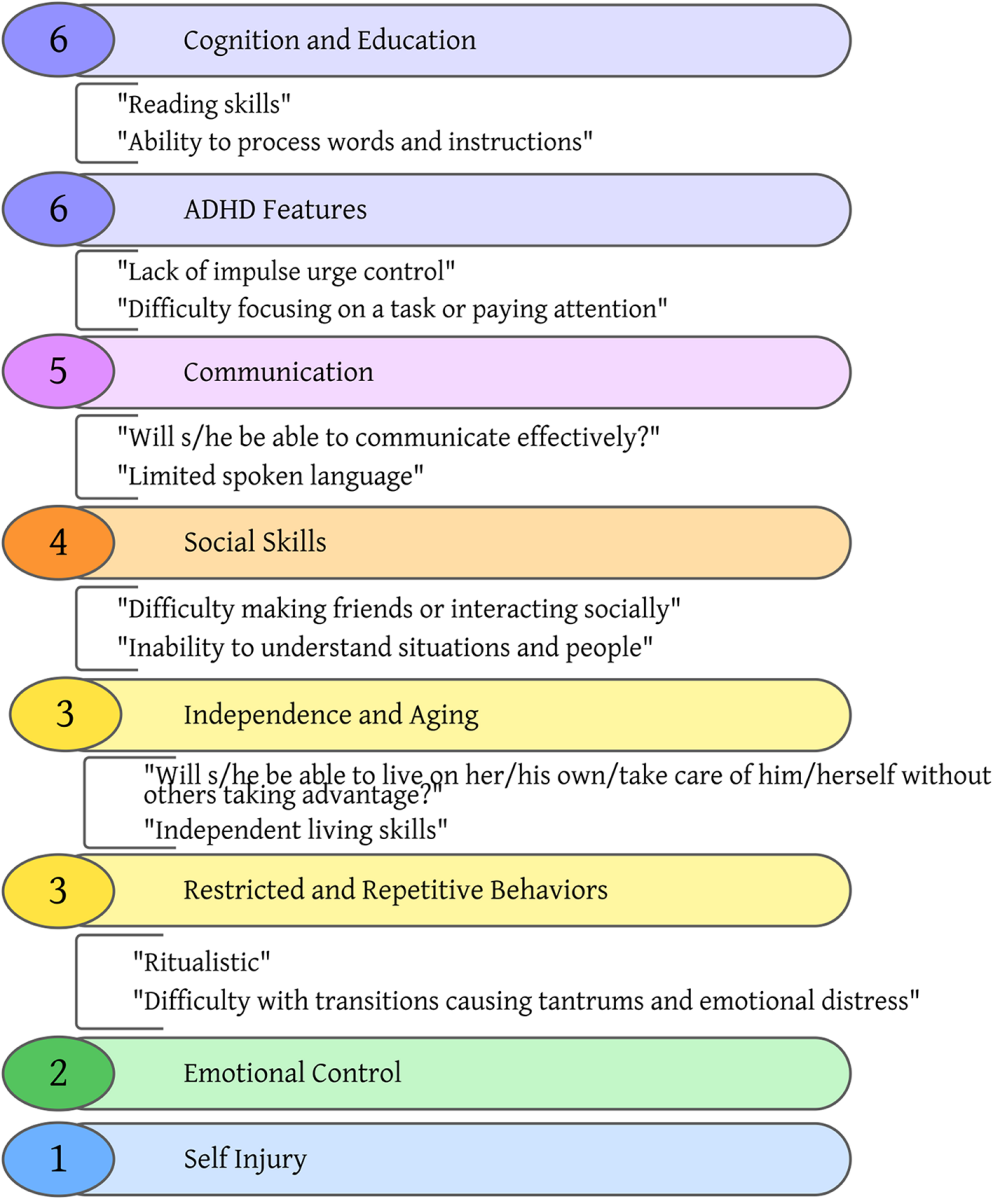
Visual Analogue Scale Caregiver Concerns *Legend*: The eight categories of concern accompanied by the number of caregivers citing that concern in the circles to the left and direct quotes from caregiver report. Colors and order of the list indicate the concern frequency

Caregiver reports on the VAS identified eight categories of concern. Each caregiver reported three concerns, totaling 30 reports (Fig. 2). The most frequently reported concerns were cognitive and educational ability and ADHD features totaling 6 responses each. A number of behaviors were reported as top concerns including communication, social skills, independence, RRBs, and self-injury. Figure 2. Visual Analogue Scale Results.

Using the CFQL, quality of life was assessed in 8 categories. Scores in each subdomain ranged from 1 to 5 and were calculated as a within-participants average for each of 3 or 4 questions in that subdomain. Based on overall average scores in each category, where lower score indicate less strain, caregivers rated Relationship Quality of Life highest (M = 1.95) followed by Financial (M = 2.40), Caregiver (M = 2.65), Social Network (M = 2.65), Coping (M = 2.70), Child (M = 2.85), Changes to Quality of Life (M = 2.94), and Family Quality of Life (3.53). The questions indicating the most strain, were the participant requiring reminders to complete everyday tasks (4.1 ± 0.99), adding stress to home life (4.1 ± 1.29), and limiting the family from participating in social activities (3.6 ± 1.17).

### Comparison to SHANK3 cohort

Compared to an age-matched group of individuals with PMS, standard scores on all Vineland-3 domains were significantly higher in the SHANK2 cohort (Table 7). The SHANK2 cohort also had smaller ranges of scores on adaptive functioning than the PMS cohort.

**Table 7:** SHANK2/PMS adaptive behavior comparison

Domain	SHANK2 (*n* = 10)	PMS (*n* = 63)	t-test *p* value
	Mean (SD)		
Composite	59.1 (15.34)	44.1 (17.6)	0.017*
Communication	47.8 (12.7)	35.1 (17.6)	0.019*
Daily Living Skills	65.0 (18.37)	46.8 (19.1)	0.015*
Socialization	63.3 (18.84)	46.5 (20.4)	0.02*
Motor	76.0 (7.04)	50.1 (20.5)	< 0.001**

*p <.05, ** *p* <.01

## Discussion

Here we present the first prospective evaluation of a cohort of individuals with SHANK2 disorder. The battery was comprised of caregiver interviews with a psychiatrist, psychologists, and a genetic counselor, caregiver forms, and review of records. Given the rarity of the syndrome, all data was collected remotely to enable enrollment across geographic regions. The comprehensive battery measured developmental level, psychiatric history, medical history, and behavioral features, taking guidance from the standards for phenotyping a new rare genetic syndrome set forth in AlMail and colleagues [45].

Results revealed some delays in motor and language milestones. Varying levels of expressive language were reported with seven of ten participants having a minimum of phrase speech by approximately 5.5 years old; the remaining three participants were all younger than this age and therefore their developmental trajectory is not yet known. Importantly, and in contrast to PMS, there was no reported loss of skills, with the exception of toileting in one participant at the age of 24, which was attributed to lack of structure during the COVID-19 pandemic.

Results from the DP-4 indicate relatively preserved Physical development, similar to results from the Early Skill interview and Vineland-3 Motor domains, however, all scores remained below age expectations. Past literature has found that motor skills are more impaired in individuals with single gene conditions than idiopathic autism [59]. While motor skills were less impaired than other areas in this cohort, the level of delays here are consistent with monogenic conditions associated with neurodevelopmental disorders. The other domains (Communication, Cognition, Social-Emotional, Adaptive) revealed scores approximately 2 standard deviations below the population mean, falling in the significantly impaired range. On the Vineland-3, Communication was a relative weakness, with average scores a full standard deviation lower than the other domain scores, reflecting slightly different aspects of communication compared to the DP-4, and mirroring results of previous SHANK2 literature [43–44]. Within the Communication domain, receptive language was reported as significantly better developed as a cohort than expressive language, suggesting the participants can understand more than they can express verbally. However, there were two participants who did not show this discrepancy in scores, and one who had significantly better expressive than receptive language, indicating variability in language skills within our sample. Overall, Vineland-3 Composite scores, on average, fell between 2 and 3 standard deviations below the population mean. Though cognitive abilities and ID status were not directly assessed, it is estimated that based on the adaptive behavior measures, most participants are likely to meet criteria for ID.

In terms of neurodevelopmental, psychiatric, and behavioral features, 90% of participants carried a formal diagnosis of autism, replicating the prevalence of the two previous SHANK2 studies [43–44]. Whether the rate of autism in SHANK2 disorder is truly this high, or if this represents a sample bias given autism is often a reason for referral for genetic testing, is unclear. Future studies with larger cohorts, recruited from a variety of sources, may help clarify autism prevalence in this disorder. The CBCL captured withdrawn behaviors well. Conversely, Social Withdrawal behaviors were generally not endorsed on the ABC. Restricted and repetitive behaviors were endorsed with the RBS-R, picking up many symptoms, most notably stereotypic behaviors. Sensory behaviors were also commonly endorsed using the SAND interview and the Sensory Profile. The SAND identified a pattern of higher seeking and hyperreactivity compared to hyporeactivity, results that are opposite to those found in individuals with PMS (SHANK3) [20]. Symptoms of ADHD were commonly reported, and five participants carried a formal diagnosis of ADHD. The CBCL captured attention problems well although items on the ABC Hyperactivity domain were endorsed at a lower rate than in the psychological evaluation given the high rate of ADHD in the cohort, again indicating the CBCL may be performing better than the ABC in this cohort. Though most caregiver surveys chosen for this study have been used and validated in IDD populations, some do not always perform well [60–62]. The measures here seemed to perform well, possibly due to the mild-to-moderate range in intellectual delays estimated in this cohort compared to more severe delays seen in other disorders. In addition, though ADHD features were a commonly reported top concern, only one participant was currently receiving medication for ADHD symptoms; this lower-than-expected use of medication should be explored in future studies.

In terms of medical history, commonly reported features mirrored those of many genetic neurodevelopmental disorders such as hypotonia, recurrent ear infections, and gastrointestinal abnormalities [18, 46, 63–66]. Overall, the cohort was not described as medically complex. Notably, unlike other genetic syndromes, including PMS, no participants had epilepsy. There was one febrile seizure reported and no other seizures reported. No similar facial dysmorphic features were observed on examination. The only relatively consistent finding was a high frontotemporal hairline in four participants.

When comparing this cohort to an age-matched sample of PMS participants, the Vineland-3 was used as an overall proxy of functioning. Individuals with SHANK2 disorder had significantly higher adaptive functioning compared to those with PMS. Though these genes belong to the same family and hold similar roles as scaffolding proteins, results from this study suggest loss of *SHANK3* may be more detrimental to human development than *SHANK2*. Larger, studies are necessary to replicate this exploratory analysis.

This study was limited by its remote nature, where participants were not assessed directly. Cognitive testing would be a valuable addition to future studies. As with all studies involving genetic disorders, sample bias is at play, where often only individuals who present with severe developmental delays are referred for genetic testing. Individuals with more mild features may not be referred for genetic testing, and therefore, results may not fully capture the full spectrum of SHANK2 disorder. Similarly, the prevalence of ASD reported in this cohort may be higher because the presence of ASD resulted in obtaining genetic testing. Therefore, there was likely a selection bias based on this. The wide age range is another limitation of this study and age effects are difficult to capture in such a small cohort. All participants were white with constrained racial and ethnic diversity, which further limits generalizability of results. Additionally, while we predicted a 50/50 sex ratio, there were more females than males in this cohort. We do not believe this is due to an underlying sex difference in SHANK2 disorder, rather an outcome of a small study. This study also did not directly assess autism in this sample, which would have required an in-person visit. Therefore, the conclusions drawn about autism symptomology are based upon parent reporting rather than clinician observation and may be biased. Finally, the comparison of SHANK2 to PMS was limited in its scope, comparing only the Vineland-3. The addition of cognitive measures in the SHANK2 cohort would allow for more robust conclusions to be drawn comparing these two genetically related groups. Future research should be conducted in person to further assess autism symptoms in SHANK2 disorder and how they differ from idiopathic autism using gold-standard diagnostic assessments.

## Conclusions

Overall, SHANK2 disorder is associated with developmental and adaptive functioning impairments indicative of a likely high rate of ID, high rates of autism, including sensory symptoms and repetitive behaviors, and ADHD, all of which were also top caregiver concerns. Nonspecific medical comorbidities were reported but individuals were not described as medically complex or requiring high levels of medical interventions. Future studies should examine larger cohorts, capture longitudinal data, and utilize in-person assessments.

## Data Availability

The datasets generated during the current study are available from the corresponding author on reasonable request and IRB approval.

## Abbreviations

ABC: Aberrant Behavior Checklist
ABCL: Adult Behavior Checklist
ADHD: Attention-Deficit/Hyperactivity Disorder
ASD: Autism Spectrum Disorder
CBCL: Child Behavior Checklist
CDC: Center for Disease Control
CFQL: Child and Family Quality of Life, Second Edition
DP-4: Developmental Profile, Fourth Edition
DSM-5: Diagnostic and Statistical Manual of Mental Disorders, Fifth Edition
ID: Intellectual Disability
IDD: Intellectual and Developmental Disability
NMDAR: N-methyl-D-aspartate glutamate receptor
OCD: Obsessive Compulsive Disorder
PMS: Phelan McDermid syndrome
RBS-R: Repetitive Behavior Scale, Revised
RRB: Ritualistic and Repetitive Behaviors
SAND: Sensory Assessment for Neurodevelopmental Disorders
SP: Sensory Profile Caregiver Questionnaire
VAS: Visual Analogue Scale
Vineland-3: Vineland Adaptive Behavior Scales, Third Edition
